# The association between future self-continuity and problematic mobile video gaming among Chinese college students: the serial mediation of consideration of future consequences and state self-control capacity

**DOI:** 10.1186/s40359-023-01256-6

**Published:** 2023-08-08

**Authors:** Junxian Shen, Jiansong Zheng, Tao Zhang

**Affiliations:** 1 Faculty of Humanities and Social Sciences, Macao Polytechnic University, Macao, People’s Republic of China; 2 Wuhan Sports University, Wuhan, Hubei People’s Republic of China

**Keywords:** Problematic mobile video gaming, Future self-continuity, Consideration of future consequences, State self-control capacity

## Abstract

**Background:**

To explore the relationship between future self-continuity and problematic mobile video gaming among Chinese college students and to examine the serial mediation of consideration of future consequences and state self-control capacity on the association between future self-continuity and problematic mobile video gaming, based on Identity-Based Motivation Theory.

**Methods:**

The Problematic Mobile Video Gaming Scale, Future Self-continuity Scale, Consideration of Future Consequences Scale, and Short Version of State Self-control Capacity Scale were administered to a sample comprising 800 college students (338 males accounting for 42.3%). Multivariate analysis and latent variables analysis were utilized to explore the separate mediating role consideration of future consequences and state self-control capacity played in the association between future self-continuity and problematic mobile video gaming, and their serial mediation also was investigated. The Bootstrap method was employed to test the significance of these mediation effects.

**Results:**

The negative association between future self-continuity and problematic mobile video gaming was moderately found. Students with increased consideration of future consequences from higher levels of future self-continuity have decreased their problematic mobile video gaming. Future self-continuity significantly positively predicted state self-control capacity, which in turn significantly negatively predicted problematic mobile video gaming. The serial mediation was also found.

**Conclusion:**

The findings revealed why differences in identification with the current and future selves become influencing factors in problematic mobile video gaming. This study observed the mediating role that consideration of future consequences and state self-control capacity play in the association between future self-continuity and problematic mobile video gaming.

## Background

Problematic mobile video gaming (PMVG) refers to the phenomenon in which the users of mobile digital devices are strongly dependent on mobile video games, and play mobile video games repeatedly over a long period of time [1]. Previous research found that problematic online gaming was positively associated with mental health problems such as social anxiety, depression, and dependence [2–4]. China’s size of mobile phone online game users has reached about 521 million, accounting for 48.9% of mobile phone netizens in 2022 [5]. With the popularity of mobile phones and tablet computers, mobile video games have become common entertainment for college students [6, 7]. It is necessary to address the potential problematic mobile video gaming among college students.

Existing literature remains lacking on the association between problematic mobile video gaming and future self-continuity and the corresponding potential mediation mechanisms. Future self-continuity refers to the psychological connection between an individual’s current self and future self [8]. Future self-continuity affects individuals’ intertemporal decision-making, mainly in terms of time discounting [9, 10]. People with lower levels of future self-continuity are more concerned about current benefits and losses than the future [9]. Individuals with higher levels of future self-continuity are more likely to view their future selves as their current selves, and they will place more importance on future gains and losses, by reducing time discounting in intertemporal decision-making [11, 12]. Mobile video games can bring immediate rewards to gamers, and make them procrastinate by increasing their time discounting for achieving long-term goals [11, 13, 14]. Mobile video games can relieve college students’ stress and make them relaxed, but excessive uses of mobile video games would increase the uncertainty about their future [15]. Future self-continuity may be able to be negatively associated with problematic mobile video gaming among college students.

This view is supported by Identity-Based Motivation Theory (IBM), which assumes that an individual’s self-concept is dynamically constructed, consisting of building the current and future self [16]. When people perceive their current tasks to be beneficial for the construction of their future identities, they will actively work to overcome the current problems in order to complete the tasks [16–18]. College students who aspire to pursue certain professions will strengthen their accessibilities to the occupations by actively participating in the relevant trainings. When college students’ perceptions of their future identities conflict with their current task, they may perceive that the current difficulties are not worth overcoming, and the idea “this is not for me” may appear in their minds resulting in refusing potential challenges from the current task [19]. College students with lower degrees of future self-continuity are more likely to participate in problematic mobile video gaming. Based on this, we developed a hypothesis that

- H1: Future self-continuity negatively predicts problematic mobile video gaming among college students.

Consideration of future consequences means that individuals’ present decision-making principles are based on current profits or future gains [20]. The General Aggression Model suggests that decision-making criteria are influenced by the interaction between perception and consideration of future consequences [21]. People with higher consideration of future consequences consider more future implications of their current behavior [9, 10]. In this vein, college students with higher levels of consideration of future consequences are more aware of the disadvantages of problematic mobile video gaming, and they may be able to readily demonstrate an immediate cessation of mobile video gaming [22]. College students with lower degrees of consideration of future consequences may indulge in mental satisfaction from mobile video games, likely resulting in their possible problematic mobile video gaming [23, 24]. Previous studies have found that future self-continuity among college students positively correlates with their consideration of future consequences [9, 15, 25]. Undergraduate students who score low on future self-continuity tend to view their future selves as strangers, and manifest less concern for that selves’ benefits [15]. In contrast, college students with high levels of future self-continuity value their own development in the future, and may consider more about the future consequences of their current actions [26]. In this context, university students with higher consideration of future consequences from increased levels of future self-continuity may show less problematic behaviors in mobile video gaming. Based on this, we proposed the hypothesis that

- H2: Future self-continuity positively predicts consideration of future consequences, which in turn negatively predicts problematic mobile video gaming.

Self-control denotes the process by which individuals change their behavioral tendencies to achieve long-term goals [27]. State self-control capacity refers to a self-control ability that is not disturbed by environments [28, 29]. State self-control capacity is able to reduce many negative behaviors such as juvenile delinquency, deceptive behavior, and aggression [16, 30]. Previous surveys among Chinese college students suggested that state self-control capacity was effective in reducing mobile phone dependence [31, 32]. College students’ state self-control capacity may be able to help them overcome their phone dependence including problematic mobile video gaming [33, 34]. It has been found that college students with higher levels of future self-continuity were more likely to have stronger state self-control capacity [35]. Future self-continuity functions to enhance self-control and shift students’ attention from short-term enjoyment from online games in their daily lives to long-term goals such as career pursuits after their graduation [15]. State self-control capacity represents a psychological resource to help students reach their desired future selves [26, 35]. In this context, state self-control capacity may play a mediating role in the association between future self-continuity and problematic mobile video gaming. Based on this, we proposed the hypothesis that

- H3: Future self-continuity positively predicts state self-control capacity, which in turn negatively predicts problematic mobile video gaming.

IBM theory suggests that individuals’ identity-based motivation drives them to devote limited time and energy to their identity-consistent behaviors [16, 36]. Identifications with certain future identities allow individuals to perceive their reachable future [37]. If people are aware that current tasks are meaningful for future developments, they will strive to complete the tasks and overcome the difficulties during the tasks through their state self-control capacity [38, 39]. RM Adelman, et al. [15] pointed to a positive association between consideration of future consequences and self-control. Students’ directing attention away from their present towards their future allows them to effectively exert self-control to regulate their behaviors for the attainment of beneficial outcomes, especially when they consider more future consequences currently [15, 29]. According to IBM theory, college students’ clear perceptions of their careers after their graduation may actively mobilize their consideration of future consequences, and then they will strive hard for beneficial future results with their higher levels of state self-control capacity, resulting in lower levels of problematic mobile video gaming [16]. Based on this, we proposed the hypothesis that

- H4: Consideration of future consequences and state self-control capacity play a serial mediating role in the relationship between future self-continuity and problematic mobile video gaming.

Gender differences in problematic online gaming have been consistently found by scholars [19, 40, 41]. Specifically, males or boys show higher risks of problematic online gaming, and may exhibit symptoms such as anxiety and depression [42, 43]. However, the gender-specific factor differs regarding time playing, social motives, and personality [44]. Boys rather than girls spend more time on online games, however, which is positively associated with their pro-social behaviors [45]. While RA Desai, et al. [45] argued that more aggressive girls were attracted to gaming. Boys love action and competitive games such as massively multiplayer online role-playing games while girls prefer casual games mainly for interpersonal motivation [44]. In this context, males’ and females’ perspectives on their future selves, as a type of personality, may have different impacts in their gaming behaviors, and even problematic mobile video gaming.

Students who are the only child in their families positively correlated with their educational resources and the expectations that they may receive from their parents, which may have a negative impact on their problematic online gaming, future considerations, and self-control capacity [46, 47]. Individuals in early grades have more free time, likely undeveloped specific goals, and are more susceptible to the temptation of problematic video online games [48]. Students from urban rather than rural areas may have more social resources in line with careers after their graduation, and possibly show higher consideration of future consequences [49, 50], higher levels of self-control capacity [32, 34], and lower prevalence of problematic video gaming [51, 52].

The contributions of this study are the exploration of the association between future self-continuity and problematic mobile video gaming, and the serial mediation effect of this relationship. In addition, we investigated the effects of demographic information on gaming disorder, such as gender differences in problematic mobile video gaming. According to the empirical evidence, theoretical contributions and practical guidance were elaborated to overcome the potential problematic mobile video gaming of university students in the Discussion section.

**Fig. 1:**
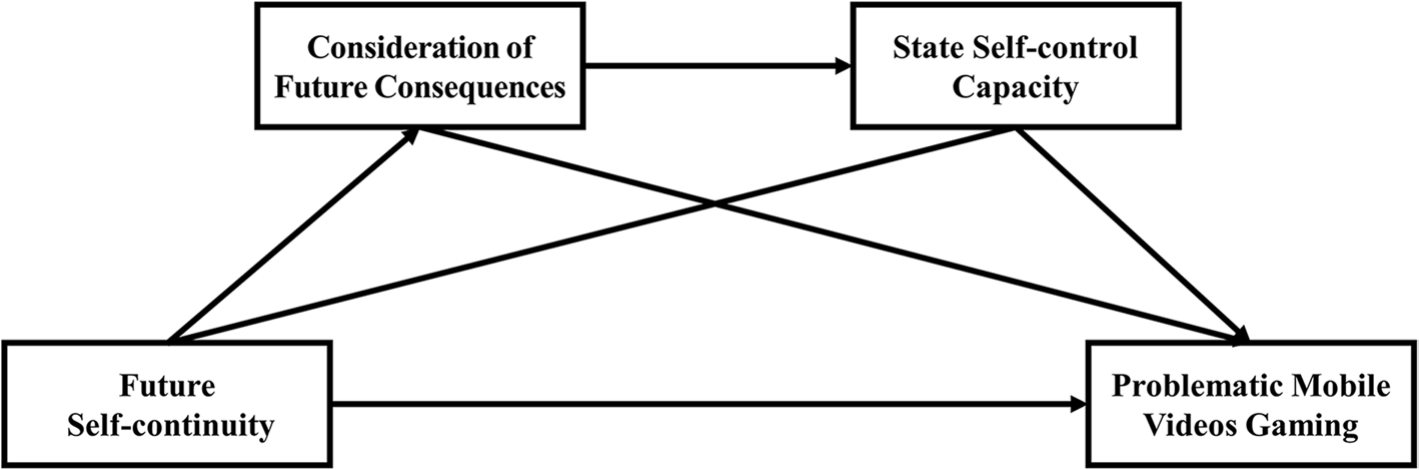
Research framework

According to IBM theory, the extent to which identities of college students after their graduation can correlate with their levels of consideration of future consequences and state self-control capacity [16]. Problematic mobile video gaming may be a roadblock in their pursuit of future identities [53, 54]. We developed mediation models with problematic mobile video gaming as the dependent variable, future self-continuity as the independent variable, and consideration of future consequences and state self-control capacity as mediating variables. The serial mediation mechanism of consideration of future consequences and state self-control capacity on the relationship between future self-continuity and problematic mobile video gaming was investigated. The specific research framework is shown in Fig. 1.

## Methods

### Sampling and procedure

We utilized a random sampling method for recruiting participants. All participants signed the informed consent form before completing the questionnaire. A questionnaire survey was conducted among freshmen, sophomores, and juniors in four universities in Wuhan, China. A total of 900 college students volunteered to participate in the survey. The validity of these questionnaires was checked by three indicators, including unlimited item scores, missing demographic information, and overfilling time. When a participant ignored any items, or spent either less than 30 s or more than 10 min filling in the questionnaire, the corresponding observation would be deleted. Finally, 800 usable questionnaires were analyzed.

SPSS 21.0 and Process 3.0 were applied [55]. Correlation and regression analyses were performed. Sobel tests with the Bootstrap method were applied to estimate 95% confidence intervals for mediating effects by randomly sampling 5000 Bootstrap samples.

### Measures

Future self-continuity (FSC) was measured by a Chinese version of Future Self-Continuity Scale (FSCS) [56]. The scale contains 10 items and each item is rated on a 6-point Likert scale. The scale includes three domains: 4 items on the similarity subscale (e.g. “How similar are you now to what you will be like 10 years from now?”), ranging from 1 (completely different) to 6 (exactly the same); 3 items on the vividness subscale (e.g. “How vividly can you imagine what you will be like in 10 years from now?”), ranging from 1 (not at all) to 6 (perfectly); 3 items on the positivity subscale (e.g. “Do you like what you will be like 10 years from now?”), ranging from 1 (not at all) to 6 (perfectly). Higher scores indicated higher degrees of respondents’ FSC.

Consideration of future consequences (CFC) was assessed by Consideration of Future Consequences Scale (CFCS) [20]. The scale contains 12 items, which were answered using a 5-point Likert scale ranging from 1 (extremely uncharacteristic) to 5 (extremely characteristic). Questions 3, 4, 5, 9, 10, 11, and 12 were reverse scored. Higher scores indicate higher levels of respondents’ CFC. Sample items included: “I consider how things might be in the future, and try to influence those things with my day to day behavior.” and “I am willing to sacrifice my immediate happiness or well-being in order to achieve future outcomes.”.

State self-control capacity (SSCC) was measured by a Chinese revision of State Self-Control Capacity Scale (SSCCS) [57]. The scale was simplified to 10 items with questions 3, 6, 9, 10, 11, 12, 13, 15, 16, and 18 in the full scale. The simplified version also had good reliability and validity [34]. Each item is rated on a 7-point Likert scale, ranging from 1 (totally disagree) to 7 (totally agree). All items were reverse scored except the 5^th^ and 8^th^ questions of the simplified version. Higher scores indicate higher degrees of respondents’ SSCC. Sample items included: “I need something pleasant to make me feel better.” and “I feel drained”.

Problematic mobile video gaming (PMVG) was assessed by the Problematic Mobile Video Gaming Scale (PMVGS) [1]. The scale contains 11 items. Each item is rated on a 5-point Likert scale, ranging from 1 (never) to 5 (very often). Higher scores indicate respondents’ worse situation of PMVG. The scale contains three domains: 4 items on the withdrawal symptoms subscale (e.g. “During the last year, have you felt miserable when you were unable to play mobile video games or played less than usual?”); 4 items on the mood modification subscale (e.g. “During the last year, have you played mobile video games to feel better?”); 3 items on conflict subscale (e.g. “During the last year, have you ever jeopardized your school or work performance because of playing mobile video games?”).

Demographic variables *Gender* was coded as “male: 1, female: 0”. *Student’s place of origin* was an ordinal variable revealing place attributes of students before rolling in their universities and was coded as “rural: 1, urban: 2, city: 3”. *Only child* referred to whether the student is the only child in a family and was coded as: “yes: 1, no: 0”. *Grade* was coded as “freshman: 1, sophomore: 2, junior: 3”.

## Results

### Reliability test results

Cronbach *α* values of scales were calculated to ensure the reliability of the data [58]. The results showed that the Cronbach *α* values of future self-continuity, consideration of future consequences, state self-control capacity, and problematic mobile video gaming in this study were 0.753, 0.773, 0.786, and 0.901, respectively. All Cronbach *α* values were more than 0.7, showing good reliability [59]. Therefore, the survey data is suitable to investigate the mediating effects of the variables.

### Test for common method bias

Because of using questionnaires, it was necessary to test the common method bias [60]. The test for common method biases was conducted by using Harman’s single-factor test, in which all items of the scales were included for factor analysis [60, 61]. The results showed that there were 10 factors with their characteristic root greater than 1. The first factor explained only 18.92% of the variance and did not reach the threshold of 40%, indicating that there were no serious common method biases.

### Multivariate analysis results

Descriptive statistics and results of correlation analysis were shown in Table 1. Correlation analysis showed that *Gender* was associated with PMVG (*ρ* = 0.163, *p* < 0.001), indicating that male college students were more likely to have PMVG than female college students. *Grade* and *Only Child* were not significantly correlated with the other variables in the study. *Student’s Place of Origin* showed positive correlations with FSC and CFC (*ρ* = 0.174, *p* < 0.001; *ρ* = 0.164, *p* < 0.001, respectively), indicating that university students coming from urban areas reported higher degrees of FSC and CFC, respectively.

**Table 1:** Means, standard deviations and correlation coefficients of the variables

Variables	M ± SD	1	2	3	4	5	6	7
1. Gender (1 = male)	42.3%							
2. Only Child (1 = Yes)	40.8%	-0.140^***^						
3. Grade		0.037	-0.012					
Freshmen	52.9%							
Sophomores	31.5%							
Juniors	15.6%							
4. Student’s place of origin (1 = urban)	66.5%	-0.090^*^	0.366^***^	-0.002				
5. FSC	29.5 ± 6.0	-0.046	-0.041	-0.070	0.174^***^			
6. CFC	38.7 ± 6.5	0.016	-0.043	0.025	0.164^***^	0.168^***^		
7. SSCC	41.3 ± 7.9	0.052	-0.014	-0.020	0.068	0.283^***^	0.376^***^	
8. PMVG	28.3 ± 9.3	0.163^***^	-0.053	0.061	-0.016	-0.214^***^	-0.262^***^	-0.472^***^

1. ***, *p* < 0.001; **, *p* < 0.01; *, *p* < 0.05

2. *N* = 800

3. FSC means Future Self-continuity; CFC means Considerations of Future Consequences; SSCC means State Self-control Capacity; PMVG means Problematic Mobile Videos Gaming

4. Percentages were reported for the categorical variables including Gender, Only Child, Grade, and Student’s Place of Origin

There was a significant positive correlation between FSC and CFC as well as FSC and SSCC, respectively (*ρ* = 0.168, *p* < 0.001; *ρ* = 0.283, *p* < 0.001) and a significant negative correlation between FSC and PMVG (*ρ* = -0.214, *p* < 0.001); CFC was significantly positively related to SSCC (*ρ* = 0.376, *p* < 0.001) and was significantly negatively correlated to PMVG (*ρ* = -0.262, *p* < 0.001); There was a significantly negative relationship between SSCC and PMVG (*ρ* = -0.472, *p* < 0.001).

Using model 6 in Process 3.0 plugin of SPSS, the serial mediating analysis was tested by controlling for all demographic variables. All variables were normalized. The results were presented in Table 2. It showed that FSC significantly positively predicted CFC (*β* = 0.148, *p* < 0.001). FSC and CFC significantly positively predicted SSCC, respectively (*β* = 0.233, *p* < 0.001; *β* = 0.342, *p* < 0.001). FSC, CFC, and SSCC significantly negatively predicted PMVG, respectively (*β* = -0.070, *p* = 0.031; *β* = -0.098, *p* = 0.003; *β* = -0.426, *p* < 0.001). The direct negative effect of FSC on PMVG was significant (*β* = -0.204, *p* < 0.001). Hypothesis 1 was tested.

**Table 2:** Results of regression analysis

	CFC		SSCC		PMVG		PMVG	
	*β*	S.E	*β*	S.E	*β*	S.E	*β*	S.E
FSC	0.148^***^	0.035	0.233^***^	0.033	-0.070^*^	0.032	-0.204^***^	0.035
CFC			0.342^***^	0.033	-0.098^**^	0.033		
SSCC					-0.426^***^	0.034		
Gender	0.021	0.071	0.126	0.065	0.356^***^	0.062	0.297^***^	0.070
Only child	-0.037	0.076	-0.014	0.070	0.074	0.067	0.086	0.075
Grade	0.046	0.047	-0.019	0.043	0.058	0.041	0.055	0.046
Student’s place of origin	0.169^***^	0.044	-0.037	0.041	0.014	0.039	-0.011	0.044
R	0.221		0.442		0.521		0.263	
F	8.121^***^		48.279^***^		59.247^***^		19.777^***^	

1. ***, *p* < 0.001; **, *p* < 0.01; *, *p* < 0.05

2. *N* = 800

3. *FSC* means Future Self-continuity, *CFC* means Considerations of Future Consequences, *SSCC* means State Self-control Capacity, *PMVG* means Problematic Mobile Videos Gaming

Table 2 also reported the impacts of demographic variables on the main variables. Gender did not saliently predict CFC and SSCC (*β* = 0.021, *p* = 0.766; *β* = 0.065, *p* = 0.055), but significantly positively predicted PMVG (*β* = 0.356, *p* < 0.001 in the model with mediating variables as the input variables; *β* = 0.297, *p* < 0.001 in the model without mediating variables as the independent variables). Males rather than females were highly likely to show PMVG, and there were no significant gender differences in CFC and SSCC. No empirical evidence supported significant predictive relations between students’ sibship and main variables including CFC, SSCC, and PMVG (*β* = -0.037, *p* = 0.629; *β* = -0.014, *p* = 0.839; *β* = 0.070, *p* = 0.270). There were insignificant predictive associations between students’ grades and variables including CFC, SSCC, and PMVG (*β* = 0.046, *p* = 0.323; *β* = -0.019, *p* = 0.653; *β* = 0.043, *p* = 0.158). Student’s place of origin significantly positively predicted their degrees of CFC (*β* = 0.169, *p* < 0.001), but showed insignificant prediction for their levels of SSCC and PMVG (*β* = -0.037, *p* = 0.365; *β* = 0.014, *p* = 0.721).

The mediation effects were verified by the Bootstrapping approach. The results were shown in Table 3. The Bootstrap 95% confidence interval without value 0 means that the mediating effect is significant. The total indirect effect was significant and accounted 66.2% of the total effect. The indirect effects of the three pathways were significant. Specifically, the pathway “FSC → CFC → PMVG” was significant and its indirect effect accounted 6.9% of the total effect. Hypothesis 2 was tested. The pathway “FSC → SSCC → PMVG” was significant and its indirect effect accounted 48.5% for the total effect. Hypothesis 3 was tested. The serial mediation pathway “FSC → CFC → SSCC → PMVG” was significant and its indirect effect accounted 10.3% of the total effect. Hypothesis 4 was tested.

**Table 3:** Results of the test for mediating effects based on the Bootstrap method

Paths	Effect Sizes	S.E	95% Confidence Intervals		Mediation Proportion
			Bootstrap LLCI	Bootstrap ULCI	
Total indirect effects	-0.135	0.022	-0.181	-0.093	66.2%
Path1: FSC → CFC → PMVG	-0.014	0.006	-0.029	-0.004	6.9%
Path2: FSC → SSCC → PMVG	-0.099	0.018	-0.137	-0.065	48.5%
Path3: FSC → CFC → SSCC → PMVG	-0.021	0.006	-0.035	-0.010	10.3%

*FSC* means Future Self-continuity, *CFC* means Considerations of Future Consequences, *SSCC* means State Self-control Capacity, *PMVG* means Problematic Mobile Videos Gaming

### Results of the structural equation model

In order to verify the net effects between the study variables, latent variables analysis without controls was employed to investigate the mediation pathways of these variables. Covariance based structural equation modeling (CB-SEM) was utilized to solve the model. Modification Indices (MI) were used to correlate terms or them and latent variables to improve the fitness of the model. The lavaan package in R language was employed to perform these actions [62].

**Fig. 2:**
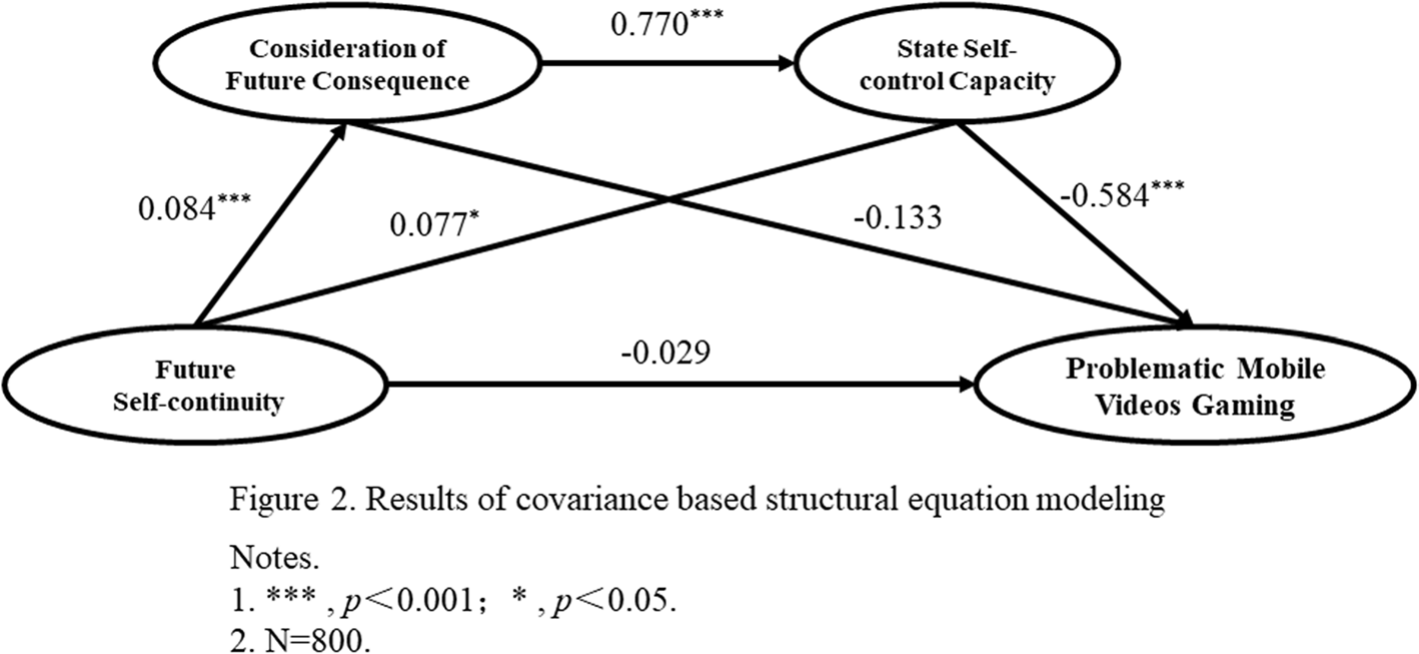
Results of covariance based structural equation modeling. Notes. 1. ***, *p* < 0.001; *, *p* < 0.05. 2. *N* = 800

Table 4 showed the fit of the structural equation model. The model fitted well with its obtained values all met the corresponding criteria [63]. Figure 2 illustrated the results of SEM. The results showed that FSC significantly predicted CFC (*β* = 0.084, *p* < 0.001). FSC and CFC significantly predicted SSCC (*β* = 0.077, *p* = 0.013; *β* = 0.770, *p* < 0.001). FSC and CFC did not have significant effects on PMVG (*β* = -0.029, *p* = 0.307; *β* = -0.133, *p* = 0.113), but SSCC significantly predicted PMVG (*β* = -0.584, *p* < 0.001). Hypothesis 1 was not supported.

**Table 4:** Fitness of the structural equation model

Type	Indicator	Abbreviation	Obtained value	Acceptable threshold value
Normed fit index	*χ* ^2^(714)	-	1.553	< 3
	Root mean square error of approximation	RMSEA	0.026	< 0.08
	Standardized root mean square residuals	SRMR	0.048	< 0.05
Absolute Fitness Indices	Goodness-of-fit Index	GFI	0.938	> 0.90
	Adjusted Goodness-of-fit Index	AGFI	0.918	> 0.90
Incremental Fit Measures	Tucker-Lewis Index	TLI	0.964	> 0.90
	Normed Fit Index	NFI	0.925	> 0.90
	Non-normed fit index	NNFI	0.964	> 0.90
	Comparative Fit Index	CFI	0.971	> 0.95

With the goal of verification for significant mediation effects, the Bootstrap method was utilized to run 5,000 Bootstrap re-samplings (Table 5). The results of the mediation effect test showed that the mediation pathway “FSC → CFC → PMVG” (*Effect* = -0.011, *p* = 0.120) was insignificant. Hypothesis 2 was not tested. However, we found two significant mediating paths including “FSC → SSCC → PMVG” (*Effect* = -0.045, *p* = 0.012) and “FSC → CFC → SSCC → PMVG” (*Effect* = -0.038, *p* < 0.001). The total indirect effect was also significant (*Effect* = -0.094, *p* < 0.001). Hypothesis 3 and 4 were supported again.

**Table 5:** Verification of mediation pathways based on the Bootstrap method

Mediation Pathways	Effect Sizes	S.E
Total indirect effects	-0.094^***^	0.022
Path1: FSC → CFC → PMVG	-0.011	0.007
Path2: FSC → SSCC → PMVG	-0.045^*^	0.018
Path3: FSC → CFC → SSCC → PMVG	-0.038^***^	0.010

1. ***, *p* < 0.001; *, *p* < 0.05

2. *N* = 800

3. *FSC* means Future Self-continuity, *CFC* means Considerations of Future Consequences, *SSCC* means State Self-control Capacity, *PMVG* means Problematic Mobile Videos Gaming

## Discussion

In the internet age, mobile video games have become a common way for young people to release their stresses [1, 52]. However, excessive mobile video gaming has given rise to several health-related problems including depression, anxiety, and procrastination [51, 64]. University students are the mainstay in the future labor market, and their connection between their present and future selves plays an important role in their careers [65]. University students with higher degrees of future self-continuity reported less bad behaviors such as academic delay and smoking [66, 67]. It is essential to uncover the possible elimination effects of future self-continuity on problematic mobile video gaming, and the relevant mediation mechanisms.

The negative association between future self-continuity and problematic mobile video gaming was found in the multivariable analysis with controls, although the latent variables analysis results did not report this direct effect. This may provide a direction to mitigate problematic mobile video gaming among college students. Future self-continuity emphasizes on the overlap of the current selves and future selves, and its higher level implies a high similarity between current selves and future selves [10]. When college students are able to touch their futures, their mobile video gaming addictions are likely weakened [68]. Future self-continuity may be a predictor of emotional stability, which has been shown to associate with problematic gaming behaviors [44, 69]. College students can improve their future self-continuity by writing letters for their future selves [10, 65]. Universities can also organize events to enhance university students’ vivid visual depictions of their future selves [70, 71]. On a larger scale, long-term orientation at the society level may enhance the future self-continuity of students in that society, and reduce their occurrence of problematic mobile video gaming [72].

The mediating role that consideration of future consequences plays in the association between future self-continuity and problematic mobile video gaming was found by the regression results, but insignificant in the structural equation model. University students with higher levels of future self-continuity probably consider more future consequences when they play games using their convenient mobile devices [26, 49]. In this vein, gaming behaviors bring them benefits such as relaxation rather than disadvantages such as gaming disorders [73]. However, students’ consideration of future consequences was a behavioral intention, rather than actual behavior, possibly leading to a weak significant predictive effect on problematic mobile video gaming [26, 74].

Empirical evidence supported the mediation effect of state self-control capacity on the relationship between future self-continuity and problematic mobile video gaming. The convenience of mobile devices tests college students’ self-control capacity, and university students with higher levels of future self-continuity tend to show higher degrees of state self-control capacity, and are able to end immediate rewards brought by games, which in turn they can avoid gaming addiction [75, 76]. The chain mediation pathway “future self-continuity → consideration of future consequences → state self-control capacity → problematic mobile video gaming” was tested, and can provide insights to college students, universities, and education sectors. Specifically, strengthening of future self-continuity in line with more consideration of future consequences and higher state self-control capacity, can have a positive impact on non-addiction to mobile video games [26, 49, 74].

In contrast to neuroanatomical features’ evidence that females are more likely to be addicted to online games [77], male participants reported more problematic mobile video gaming in our survey. Similarly, most studies have revealed that a greater proportion of men spend more time on games, which in turn likely predicts their problematic gaming behaviors [44, 78, 79]. Gender differences in gaming addiction need to be disaggregated [40]. Specifically, males prefer multiplayer online role-playing games, which can give them senses of satisfaction and vanity, and the potential inconsistency between the virtual and real world may contribute to their problematic gaming behaviors [44, 80]. However, girls prefer casual games for their interpersonal motives, which may predispose them to emotional issues, and the gamers are more likely to exhibit anxiety and depressive symptoms [40, 81, 82]. In conclusion, gender differences in problematic mobile video games differ likely regarding the types of games [44, 78]. Follow-up studies are necessary to consider the males’ and females’ gaming addiction caused by different forms of games.

The empirical data showed that university students who come from urban rather than rural areas consider more consequences of their future for the possible reason that urban students have greater social capitals, so they can easily consider more consequences of current behavior on themselves or their families in the future [83, 84]. However, there is no empirical evidence supporting that students from urban areas exhibited higher levels of state self-control capacity and fewer behaviors of problematic gaming, conceivably because the prevalence and cheaper price of smart communication tools eliminate possible urban–rural differences in students’ self-control capacity and problematic mobile video gaming [85, 86]. University students’ grade and attribute of the only child did not significantly affect their consideration of future outcomes, self-control capacity, and problematic mobile video gaming, possibly because China’s information development allowed individuals of different grades and with different numbers of siblings to have equal access to education and information resources, causing potential indifferent situations on problematic mobile video gaming [87, 88].

Although we obtained some beneficial conclusions, there was still a limitation in the study. The cross-sectional data used in the study made it unable to observe causality. Follow-up surveys can be considered to manipulate the factors of future self-continuity, and track the corresponding alternations in problematic mobile video gaming among college students.

## Conclusions

Based on the IBM theoretical framework, the association between future self-continuity and problematic mobile video gaming among college students was explored. The separate mediating effects and the serial mediation of consideration of future consequences and state self-control capacity in the aforementioned relation were explored. Results indicated that (i) university students’ future self-continuity negatively predicted their problematic mobile video gaming. (ii) University students’ future self-continuity positively predicted their consideration of future consequences, which in turn negatively predicted their problematic mobile video gaming. (iii) University students’ future self-continuity significantly positively predicted their state self-control capacity, which in turn significantly negatively predicted their problematic mobile video gaming. (iv) University students’ future self-continuity significantly positively predicted their consideration of future consequences and state self-control capacity, which in turn significantly negatively predicted their problematic mobile video gaming. The findings encourage college students to develop future self-continuity to curb online gaming addiction, and provide direction for universities and education sectors to prepare college students for better employments by enhancing their future self-continuity.

## Data Availability

The dataset used and analyzed during the current study is available from the corresponding author on reasonable request.

## Abbreviations

PMVG: Problematic Mobile Video Gaming
FSC: Future Self-continuity
CFC: Consideration of Future Consequences
SSCC: State Self-control Capacity
CB-SEM: Covariance based structural equation modeling
MI: Modification Indices
